# Methyl gallate

**DOI:** 10.1107/S1600536809001123

**Published:** 2009-01-14

**Authors:** Deborah Bebout, Silvina Pagola

**Affiliations:** a Department of Chemistry, College of William and Mary, PO Box 8795, Williamsburg, VA 23187-8795, USA; b Department of Physics, College of William and Mary, PO Box 8795, Williamsburg, VA 23187-8795, USA

## Abstract

The crystal structure of the title compound (systematic name: methyl 3,4,5-trihydroxy­benzoate), C_8_H_8_O_5_, is composed of essentially planar mol­ecules [maximum departures from the mean carbon and oxygen skeleton plane of 0.0348 (10) Å]. The H atoms of the three hydroxyl groups, which function as hydrogen-bond donors and acceptors simultaneously, are oriented in the same direction around the aromatic ring. In addition to two intra­molecular hydrogen bonds, each mol­ecule is hydrogen bonded to six others, creating a three-dimensional hydrogen-bonded network.

## Related literature

For natural extracts containing gallic acid methyl ester, see: Saxena *et al.* (1994 ▶); Schmidt *et al.* (2003 ▶); Hawas (2007 ▶). For studies concerning anti­oxidant activity, see: Aruoma *et al.* (1993 ▶); Schmidt *et al.* (2003 ▶); Hawas (2007 ▶). For studies concerning anti­cancer properties, see: Fiuza *et al.* (2004 ▶) and for anti­microbial properties, see: Saxena *et al.* (1994 ▶); Landete *et al.* (2007 ▶). For cocrystals containing gallic acid methyl ester, see: Sekine *et al.* (2003 ▶); Martin *et al.* (1986 ▶). Similar gallate ester conformations are found in Parkin *et al.* (2002 ▶); Okabe & Kyoyama (2002*a* ▶); Nomura *et al.* (2000 ▶); Mizuguchi *et al.* (2005 ▶). For structures with similar hydroxyl arrangements, see: Hitachi *et al.* (2005 ▶); Okabe *et al.* (2001 ▶); Okabe & Kyoyama (2002*b* ▶). For a description of the Cambridge Structural Database, see: Allen (2002 ▶).
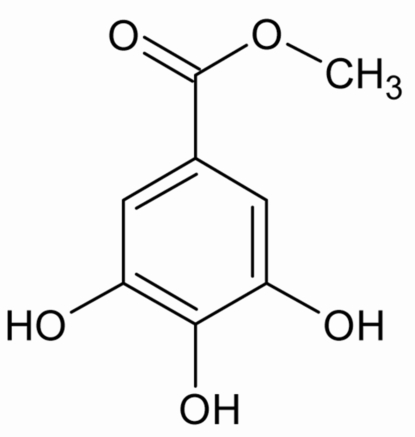


## Experimental

### 

#### Crystal data

C_8_H_8_O_5_
*M* *_r_* = 184.14
Monoclinic, 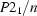

*a* = 7.6963 (2) Å
*b* = 9.9111 (2) Å
*c* = 10.5625 (2) Å
β = 95.9930 (10)°
*V* = 801.29 (3) Å^3^
*Z* = 4
Cu *K*α radiation
μ = 1.12 mm^−1^
*T* = 100 (2) K
0.31 × 0.23 × 0.21 mm

#### Data collection

Bruker SMART APEXII CCD diffractometer
Absorption correction: numerical (*SADABS*; Sheldrick, 2004 ▶) *T*_min_ = 0.723, *T*_max_ = 0.799
8192 measured reflections
1352 independent reflections
1311 reflections with *I* > 2σ(*I*)
*R*_int_ = 0.034

#### Refinement

*R*[*F* ^2^ > 2σ(*F* ^2^)] = 0.033
*wR*(*F* ^2^) = 0.093
*S* = 0.69
1352 reflections
121 parameters
All H-atom parameters refined
Δρ_max_ = 0.23 e Å^−3^
Δρ_min_ = −0.20 e Å^−3^

### 

Data collection: *APEX2* (Bruker , 2004 ▶); cell refinement: *SAINT-Plus* (Bruker, 2004 ▶); data reduction: *SAINT-Plus*; program(s) used to solve structure: *SHELXS97* (Sheldrick, 2008 ▶); program(s) used to refine structure: *WinGX* (Farrugia, 1999 ▶); molecular graphics: *ORTEP-3* (Farrugia, 1997 ▶) and *Mercury* (Macrae *et al.*, 2006 ▶); software used to prepare material for publication: *SHELXL97* (Sheldrick, 2008 ▶).

## Supplementary Material

Crystal structure: contains datablocks global, I. DOI: 10.1107/S1600536809001123/rz2286sup1.cif

Structure factors: contains datablocks I. DOI: 10.1107/S1600536809001123/rz2286Isup2.hkl

Additional supplementary materials:  crystallographic information; 3D view; checkCIF report

## Figures and Tables

**Table 1:** Hydrogen-bond geometry (Å, °)

*D*—H⋯*A*	*D*—H	H⋯*A*	*D*⋯*A*	*D*—H⋯*A*
O3—H3⋯O4	0.84	2.25	2.7075 (13)	115
O4—H4⋯O5	0.84	2.29	2.7247 (12)	112
O4—H4⋯O1^i^	0.84	2.15	2.9470 (13)	159
O3—H3⋯O1^ii^	0.84	1.99	2.7007 (12)	142
O5—H5⋯O3^iii^	0.84	1.86	2.6859 (12)	166

Symmetry codes: (i) 
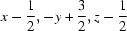
; (ii) 
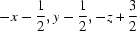
; (iii) 
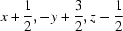
.

**Fig. 1.**
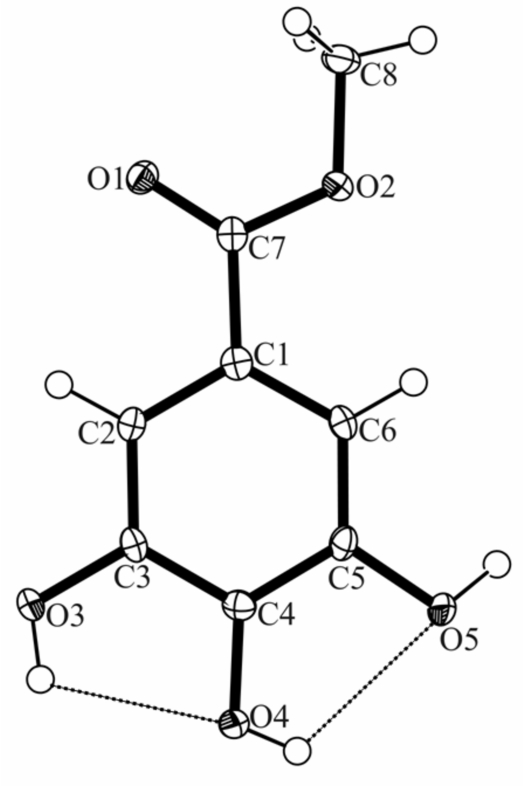
Thermal ellipsoid plot of gallic acid methyl ester with the atomic numbering scheme. Non-H atoms are represented at 50% probability level. Intramolecular hydrogen bonds are shown with dashed lines.

**Fig. 2.**
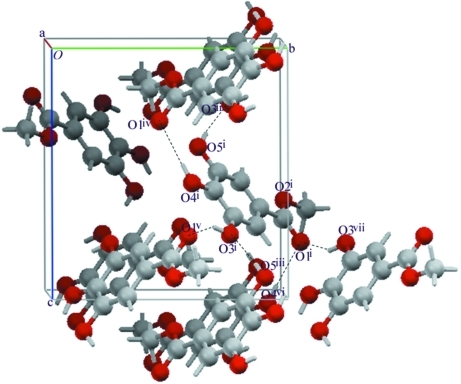
The packing of gallic acid methyl ester molecules along the a-axis showing the unit cell and the hydrogen bonded network formed (dashed lines). Symmetry codes: (i) x,y,z; (ii) 1/2 + x, 1.5 - y, -1/2 + z; (iii) -1/2 + x, 1.5 - y, 1/2 + z; (iv) -1/2 + x,1.5 - y, -1/2 + z; (v) -1/2 - x, -1/2 + y, 1.5 - z; (vi) 1/2 + x, 1.5 - y, 1/2 + z; (vii) -1/2 - x, 1/2 + y, 1.5 - z.

## supplementary crystallographic information

### Comment

Gallic acid methyl ester (I) is a polyphenolic compound present in grape seeds and other natural substrates (Saxena *et al.*, 1994; Schmidt *et al.*, 2003; Hawas, 2007). Like other polyphenols, I shows antioxidant activity (Aruoma *et al.*, 1993; Schmidt *et al.*, 2003; Hawas, 2007). Formerly used as an astringent and in opthalmology, its anticancer (Fiuza *et al.*, 2004) and antimicrobial properties (Saxena *et al.*, 1994; Landete *et al.*, 2007) have also been studied. The molecular structure of I is shown below.

The molecular geometry is as expected from chemical bond rules (Figure 1) and it shows an almost planar conformation, with maximum departures from the mean carbon and oxygen skeleton plane of 0.0343 (9) and 0.0348 (10) Å for O4 and C8, respectively. The relative positions of the carbonyl and the three hydroxyls were also observed in a cocrystal of I and 5-chloro-2-methyl-4-isothiazoline-3-one (Sekine *et al.*, 2003). Four other compounds containing a gallic acid ester moiety have crystallized in an analogous conformation (Parkin *et al.*, 2002; Okabe & Kyoyama, 2002*a*; Nomura *et al.*, 2000; Mizuguchi *et al.*, 2005). Three other planar conformations of gallic acid esters are found in the Cambridge Structural Database (Allen, 2002). I has one of these other conformations in a cocrystal with caffeine (Martin *et al.*, 1986).

Crystallized I has intra- and intermolecular hydrogen bonding. The hydroxyl H atoms bound to O3 and O4 (donors) form intramolecular hydrogen bonds to O4 and O5 (acceptors), respectively. This is shown in Figure 1. Similar hydroxyl arrangements have been reported in other gallic acid derivatives, such as gallate ester solvates (Hitachi *et al.*, 2005), a gallic acid monohydrate polymorph (Okabe *et al.*, 2001) and 2,3,4-trihydroxybenzophenone monohydrate (Okabe & Kyoyama, 2002*b*).

Gallic acid methyl ester forms a three-dimensional H-bonded network lacking significant aromatic ring stacking interactions. There is one molecule of I in the asymmetric unit. The H-bonded network is shown in Figure 2. Using the carbonyl ester oxygen O1 (acceptor) and the hydroxyl O3 and O4 (donors), each molecule is linked to another four through two O1···H3—O3, and two O1···H4—O4 H-bonds. These H-bonds are likely relatively weak due to the spacial orientation of the H atoms with respect to the lone electron pairs of O1. In addition, there are two other O5—H5···O3 H-bonds. The three hydroxyl sites are used as hydrogen bond donors and acceptors simultaneously. In the ester group, only the carbonyl oxygen is used as an H-bond acceptor.

### Experimental

Gallic acid methyl ester was commercially obtained from Sigma-Aldrich (98% purity) and used as received. The crystal structure determination was carried out from a crystal with rhombic prismatic habit selected from the powder.

### Refinement

All hydrogen atoms were observed in the Fourier difference map. However, the torsion angle for the hydroxyl H was refined from the electron density and the methyl H was positioned in idealized staggered geometry. The H atoms were refined constrained to ride on their parent C or O atoms, with *U*_iso_(H)=1.2 *U*_eq_(C) for aromatic H, and *U*_iso_(H)=1.5 *U*_eq_(C or O) for methyl and hydroxyl H, respectively.

### Crystal data

**Table:**

C_8_H_8_O_5_	*F*(000) = 384
*M**_r_* = 184.14	*D*_x_ = 1.526 Mg m^−^^3^
Monoclinic, *P*2_1_/*n*	Melting point: 474 K
Hall symbol: -P 2yn	Cu *K*α radiation, λ = 1.54178 Å
*a* = 7.6963 (2) Å	Cell parameters from 1352 reflections
*b* = 9.9111 (2) Å	θ = 6.1–67.0°
*c* = 10.5625 (2) Å	µ = 1.12 mm^−^^1^
β = 95.993 (1)°	*T* = 100 K
*V* = 801.29 (3) Å^3^	Rhombic prism, colourless
*Z* = 4	0.31 × 0.23 × 0.21 mm

### Data collection

**Table:**

Bruker SMART APEXII CCD diffractometer	1352 independent reflections
Radiation source: fine-focus sealed tube	1311 reflections with *I* > 2σ(*I*)
graphite	*R*_int_ = 0.034
ω and ψ scans	θ_max_ = 67.0°, θ_min_ = 6.1°
Absorption correction: numerical (*SADABS*; Sheldrick, 2004)	*h* = −8→9
*T*_min_ = 0.723, *T*_max_ = 0.799	*k* = −11→11
8192 measured reflections	*l* = −12→12

### Refinement

**Table:**

Refinement on *F*^2^	Primary atom site location: structure-invariant direct methods
Least-squares matrix: full	Secondary atom site location: difference Fourier map
*R*[*F*^2^ > 2σ(*F*^2^)] = 0.033	Hydrogen site location: difference Fourier map
*wR*(*F*^2^) = 0.093	All H-atom parameters refined
*S* = 0.69	*w* = 1/[σ^2^(*F*_o_^2^) + (0.0834*P*)^2^ + 0.9466*P*] where *P* = (*F*_o_^2^ + 2*F*_c_^2^)/3
1352 reflections	(Δ/σ)_max_ = 0.004
121 parameters	Δρ_max_ = 0.23 e Å^−^^3^
0 restraints	Δρ_min_ = −0.20 e Å^−^^3^
32 constraints	

### Special details

**Table:**

Geometry. All e.s.d.'s (except the e.s.d. in the dihedral angle between two l.s. planes) are estimated using the full covariance matrix. The cell e.s.d.'s are taken into account individually in the estimation of e.s.d.'s in distances, angles and torsion angles; correlations between e.s.d.'s in cell parameters are only used when they are defined by crystal symmetry. An approximate (isotropic) treatment of cell e.s.d.'s is used for estimating e.s.d.'s involving l.s. planes.

### Fractional atomic coordinates and isotropic or equivalent isotropic displacement parameters (Å^2^)

**Table:**

	*x*	*y*	*z*	*U*_iso_*/*U*_eq_	
O1	0.14003 (11)	1.05605 (9)	0.77633 (8)	0.0145 (2)	
O2	0.29090 (12)	0.99023 (9)	0.61631 (8)	0.0156 (3)	
O3	−0.38148 (12)	0.74067 (10)	0.72722 (9)	0.0189 (3)	
H3	−0.4389	0.6742	0.6963	0.028*	
O4	−0.33530 (12)	0.58249 (9)	0.52444 (9)	0.0167 (2)	
H4	−0.314	0.5462	0.4559	0.025*	
O5	−0.05442 (13)	0.61740 (10)	0.39075 (9)	0.0185 (3)	
H5	0.0127	0.6524	0.3422	0.028*	
C7	0.15489 (17)	0.98352 (13)	0.68447 (12)	0.0121 (3)	
C1	0.02736 (17)	0.87858 (13)	0.63777 (12)	0.0126 (3)	
C2	−0.11958 (17)	0.85957 (13)	0.70290 (12)	0.0126 (3)	
H2	−0.1378	0.9145	0.774	0.015*	
C3	−0.23803 (17)	0.75983 (13)	0.66260 (12)	0.0129 (3)	
C4	−0.21306 (17)	0.67861 (13)	0.55808 (12)	0.0129 (3)	
C5	−0.06683 (17)	0.69987 (13)	0.49284 (12)	0.0134 (3)	
C6	0.05370 (16)	0.79838 (13)	0.53249 (12)	0.0134 (3)	
H6	0.1539	0.8116	0.4885	0.016*	
C8	0.42258 (18)	1.09026 (14)	0.65644 (13)	0.0182 (3)	
H8A	0.5161	1.0867	0.6003	0.027*	
H8B	0.4712	1.0715	0.7441	0.027*	
H8C	0.3695	1.1802	0.6521	0.027*	

### Atomic displacement parameters (Å^2^)

**Table:**

	*U*^11^	*U*^22^	*U*^33^	*U*^12^	*U*^13^	*U*^23^
O1	0.0147 (5)	0.0147 (5)	0.0141 (5)	0.0011 (4)	0.0010 (4)	−0.0023 (4)
O2	0.0135 (5)	0.0186 (5)	0.0153 (5)	−0.0048 (4)	0.0040 (4)	−0.0030 (4)
O3	0.0171 (5)	0.0206 (5)	0.0209 (5)	−0.0067 (4)	0.0108 (4)	−0.0070 (4)
O4	0.0188 (5)	0.0180 (5)	0.0141 (5)	−0.0071 (4)	0.0058 (4)	−0.0043 (4)
O5	0.0237 (5)	0.0196 (5)	0.0140 (5)	−0.0080 (4)	0.0103 (4)	−0.0057 (4)
C1	0.0135 (6)	0.0127 (6)	0.0114 (6)	0.0019 (5)	0.0004 (5)	0.0028 (5)
C2	0.0147 (7)	0.0130 (6)	0.0104 (6)	0.0023 (5)	0.0021 (5)	−0.0002 (5)
C3	0.0127 (6)	0.0147 (6)	0.0120 (6)	0.0012 (5)	0.0041 (5)	0.0025 (5)
C4	0.0137 (6)	0.0123 (6)	0.0124 (6)	−0.0012 (5)	0.0000 (5)	0.0022 (5)
C5	0.0176 (7)	0.0129 (6)	0.0100 (6)	0.0012 (5)	0.0032 (5)	0.0002 (5)
C6	0.0127 (6)	0.0159 (7)	0.0121 (6)	0.0004 (5)	0.0040 (5)	0.0024 (5)
C7	0.0129 (7)	0.0122 (6)	0.0111 (6)	0.0037 (5)	0.0006 (5)	0.0030 (5)
C8	0.0157 (7)	0.0203 (7)	0.0185 (7)	−0.0071 (5)	0.0015 (6)	−0.0010 (5)

### Geometric parameters (Å, °)

**Table:**

O1—C7	1.2225 (16)	C1—C6	1.3988 (19)
O2—C7	1.3327 (15)	C2—C3	1.3815 (18)
O2—C8	1.4491 (16)	C2—H2	0.95
O3—C3	1.3705 (15)	C3—C4	1.3957 (18)
O3—H3	0.84	C4—C5	1.3956 (18)
O4—C4	1.3598 (16)	C5—C6	1.3813 (18)
O4—H4	0.84	C6—H6	0.95
O5—C5	1.3644 (16)	C8—H8A	0.98
O5—H5	0.84	C8—H8B	0.98
C7—C1	1.4790 (19)	C8—H8C	0.98
C1—C2	1.3965 (17)		
			
C7—O2—C8	116.16 (10)	O4—C4—C5	123.25 (11)
C3—O3—H3	109.5	O4—C4—C3	117.52 (11)
C4—O4—H4	109.5	C5—C4—C3	119.22 (12)
C5—O5—H5	109.5	O5—C5—C6	124.25 (11)
O1—C7—O2	122.91 (12)	O5—C5—C4	115.24 (12)
O1—C7—C1	124.36 (11)	C6—C5—C4	120.51 (11)
O2—C7—C1	112.72 (11)	C5—C6—C1	119.62 (12)
C2—C1—C6	120.47 (12)	C5—C6—H6	120.2
C2—C1—C7	118.29 (11)	C1—C6—H6	120.2
C6—C1—C7	121.24 (12)	O2—C8—H8A	109.5
C3—C2—C1	119.15 (12)	O2—C8—H8B	109.5
C3—C2—H2	120.4	H8A—C8—H8B	109.5
C1—C2—H2	120.4	O2—C8—H8C	109.5
O3—C3—C2	119.06 (11)	H8A—C8—H8C	109.5
O3—C3—C4	119.91 (12)	H8B—C8—H8C	109.5
C2—C3—C4	121.03 (12)		
			
C8—O2—C7—O1	−0.42 (18)	C2—C3—C4—O4	179.88 (11)
C8—O2—C7—C1	−179.68 (10)	O3—C3—C4—C5	179.65 (11)
O1—C7—C1—C2	−0.53 (19)	C2—C3—C4—C5	−0.7 (2)
O2—C7—C1—C2	178.71 (11)	O4—C4—C5—O5	0.45 (19)
O1—C7—C1—C6	−179.44 (12)	C3—C4—C5—O5	−178.97 (11)
O2—C7—C1—C6	−0.19 (17)	O4—C4—C5—C6	−179.37 (11)
C6—C1—C2—C3	0.47 (19)	C3—C4—C5—C6	1.21 (19)
C7—C1—C2—C3	−178.44 (11)	O5—C5—C6—C1	179.28 (12)
C1—C2—C3—O3	179.52 (11)	C4—C5—C6—C1	−0.92 (19)
C1—C2—C3—C4	−0.17 (19)	C2—C1—C6—C5	0.07 (19)
O3—C3—C4—O4	0.19 (18)	C7—C1—C6—C5	178.95 (11)

### Hydrogen-bond geometry (Å, °)

**Table:**

*D*—H···*A*	*D*—H	H···*A*	*D*···*A*	*D*—H···*A*
O3—H3···O4	0.84	2.25	2.7075 (13)	115
O4—H4···O5	0.84	2.29	2.7247 (12)	112
O4—H4···O1^i^	0.84	2.15	2.9470 (13)	159
O3—H3···O1^ii^	0.84	1.99	2.7007 (12)	142
O5—H5···O3^iii^	0.84	1.86	2.6859 (12)	166

Symmetry codes: (i) *x*−1/2, −*y*+3/2, *z*−1/2; (ii) −*x*−1/2, *y*−1/2, −*z*+3/2; (iii) *x*+1/2, −*y*+3/2, *z*−1/2.

## References

[bb1] Allen, F. H. (2002). *Acta Cryst.* B**58**, 380–388. 10.1107/s0108768102003890 12037359

[bb2] Aruoma, O. I., Murcia, A., Butler, J. & Halliwell, B. (1993). *J. Agric. Food Chem.* **41**, 1880–1885.

[bb3] Bruker (2004). *APEX2* and *SAINT-Plus* Bruker AXS Inc., Madison Wisconsin, U. S. A..

[bb4] Farrugia, L. J. (1997). *J. Appl. Cryst.* **30**, 565.

[bb5] Farrugia, L. J. (1999). *J. Appl. Cryst.* **32**, 837–838.

[bb6] Fiuza, S. M., Gomes, C., Teixeira, L. J., Girao da Cruz, M. T., Cordeiro, M. N. D. S., Milhazes, N., Borges, F. & Marques, M. P. M. (2004). *Bioorg. Med. Chem.* **12**, 3581–3589. 10.1016/j.bmc.2004.04.026 15186842

[bb7] Hawas, U. W. (2007). *Nat. Prod. Res.* **21**, 632–640. 10.1080/14786410701371124 17613821

[bb8] Hitachi, A., Makino, T., Iwata, S. & Mizuguchi, J. (2005). *Acta Cryst.* E**61**, o2590–o2592.

[bb9] Landete, J. M., Rodriguez, H., De las Rivas, B. & Munoz, R. (2007). *J. Food. Prot.*, **70**, 2670–2675. 10.4315/0362-028x-70.11.2670 18044455

[bb10] Macrae, C. F., Edgington, P. R., McCabe, P., Pidcock, E., Shields, G. P., Taylor, R., Towler, M. & van de Streek, J. (2006). *J. Appl. Cryst.* **39**, 453–457.

[bb11] Martin, R., Lilley, T. H., Bailey, N. A., Falshaw, C. P., Haslam, E., Magnolato, D. & Begley, M. J. (1986). *Chem. Commun.* pp. 105–106.

[bb12] Mizuguchi, J., Hitachi, A., Iwata, S. & Makino, T. (2005). *Acta Cryst.* E**61**, o2593–o2595.

[bb13] Nomura, E., Hosoda, A. & Taniguchi, H. (2000). *Org. Lett.* **2**, 779–781. 10.1021/ol9913993 10814427

[bb14] Okabe, N. & Kyoyama, H. (2002*a*). *Acta Cryst.* E**58**, o245–o247.

[bb15] Okabe, N. & Kyoyama, H. (2002*b*). *Acta Cryst.* E**58**, o565–o567.

[bb16] Okabe, N., Kyoyama, H. & Suzuki, M. (2001). *Acta Cryst.* E**57**, o764–o766.

[bb17] Parkin, A., Parsons, S., Robertson, J. H. & Tasker, P. A. (2002). *Acta Cryst.* E**58**, o1348–o1350.

[bb18] Saxena, G., McCutcheon, A. R., Farmer, S., Towers, G. H. N. & Hancock, R. (1994). *J. Ethnopharm.* **42**, 95–99. 10.1016/0378-8741(94)90102-3 8072309

[bb19] Schmidt, S., Niklova, I., Pokorny, J., Farkas, P. & Sekretar, S. (2003). *Eur. J. Lipid Sci. Technol.* **105**, 427–435.

[bb22] Sekine, A., Mitsumori, T., Uekusa, H., Ohashi, Y. & Yagi, M. (2003) *Anal. Sci. X-Ray Struct. Anal. Online*, **19**, x47–x48.

[bb20] Sheldrick, G. M. (2004). *SADABS* University of Göttingen, Germany.

[bb21] Sheldrick, G. M. (2008). *Acta Cryst.* A**64**, 112–122. 10.1107/S0108767307043930 18156677

